# Lysyl oxidase inhibition in primary myelofibrosis: A renewed strategy

**DOI:** 10.46439/stemcell.1.005

**Published:** 2020-12

**Authors:** Andrew Piasecki, Orly Leiva, Katya Ravid

**Affiliations:** 1 Department of Medicine and Whitaker Cardiovascular Institute, Boston University School of Medicine, Boston MA 02118, USA; 2 Department of Biology, Boston University, Boston, MA 02215, USA; 3 Department of Medicine, Brigham and Women’s Hospital and Harvard Medical School, Boston, MA 02115, USA

## Abstract

Primary myelofibrosis (PMF) is a type of myeloproliferative neoplasm (MPN) that portends a poor prognosis and has limited options for treatment. PMF is often driven by clonal mutations in one of three genes that regulate the JAK-STAT signaling pathway, leading to hyperactivation of this signaling pathway and over-proliferation of megakaryocytes (MKs) and their precursors. PMF presents with debilitating symptoms such as splenomegaly and weight loss. The few available treatments for PMF include a JAK2 inhibitor, ruxolitinib, which causes side effects and is not always effective. The extracellular matrix (ECM) and bone marrow (BM) microenvironment may play an important role in the pathogenesis of PMF. Lysyl oxidase (LOX), an enzyme that plays a key role in the ECM by facilitating the cross-linking of collagen and elastin fibers, has been shown to be upregulated in MKs of PMF mice and in PMF patients, suggesting its role in the progression of BM fibrosis. Recently, LOX has been identified as a potential novel therapeutic target for PMF and the development of new small molecule LOX inhibitors, PXS-LOX_1 and PXS-LOX_2, has shown some promise in slowing the progression of PMF in pre-clinical studies. Given that these inhibitors displayed an ability to target the dysregulation of the ECM via LOX inhibition, they show promise as therapeutic agents for an underappreciated aspect of PMF.

## Introduction

Primary myelofibrosis (PMF) is a myeloproliferative neoplasm (MPN) that originates from the clonal proliferation of hematopoietic stem cells and leads to progressive bone marrow (BM) fibrosis [1,2]. PMF entails a poor prognosis with limited treatment options [3]. Mutations in one of three genes that regulate the JAK-STAT signaling pathway (JAK2, MPL, and CALR) are present in 90% of PMF cases [4,5]. While mutations in MPL and CALR are found in 25% and 5% of PMF cases respectively, the most common mutation is the JAK2V617F mutation which is present in 60% of PMF cases [4,6–9]. All of these mutations result in the hyper-activation of the JAK/STAT signaling pathway, leading to increased proliferation [6–8]. All PMF cases experience dysregulation in the remodeling of the extracellular matrix (ECM), which causes increased deposition to the ECM resulting in fibrosis [10]. Additionally, PMF patients display splenomegaly and an expansion of the megakaryocyte (MK) lineage. An increase in immature, low-ploidy MKs is a hallmark of PMF [11]. The close relation between the progression of PMF and the dysregulation of the ECM prompted investigation into its causes. One factor found to be a driver of the dysregulation was an overabundance of lysyl oxidase (LOX), an enzyme known to be critical in the organization of ECM.

### Effects of Lysyl Oxidase Cross-Linking in ECM are Significant to the Onset of BM Fibrosis

**Figure 1:**
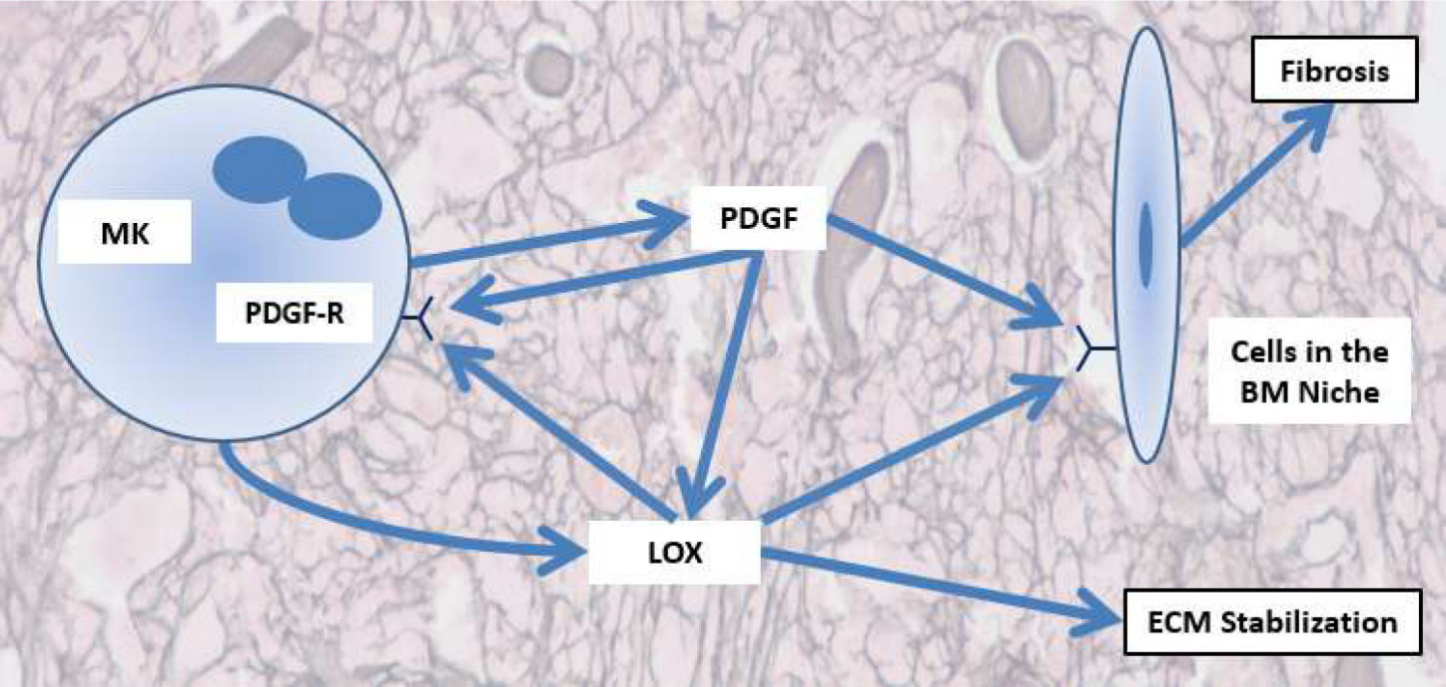
Schematic representation of the role of LOX in PMF. Adapted from Leiva et al. [10].

Lysyl oxidases are copper-dependent enzymes that cause oxidative deamination of lysine and hydroxylysine residues in the ECM structural proteins collagen and elastin [12]. More specifically, LOX oxidizes amines in lysine residues to aldehydes, which spontaneously reacts with corresponding aldehydes to form various di-, tri-, or tetrafunctional cross-links. This function allows for augmented cross-linking of collagen and elastin in the ECM, increasing its stiffness [12]. In mammals, the lysyl oxidase family is comprised of five members- lysyl oxidase (LOX), and lysyl oxidase like-1 to lysyl oxidase like-4 (LOXL1–LOXL4). Each enzyme consists of a single peptide and has a conserved C-terminal domain which performs the deamination of the lysine and hydroxylysine residues in the ECM, while N-terminal pro-region is more varied between the members. In order to perform their function of modifying collagen and elastin, lysyl oxidases are excreted into the ECM. One family member in particular, LOX, is of interest due to its expression profile in MKs. LOX expression is highest in immature MKs and is downregulated in mature MKs [11,13,14]. PMF patients have an increased number of immature MKs and increased MK LOX expression has been shown in human patients and mouse PMF models [11,13,14]. This upregulation of LOX in PMF leads to increased cross-linking and rigidity in the ECM, which contributes to the development of BM fibrosis. As illustrated in Figure 1, besides its role in modifying the ECM, LOX has also been shown to affect platelet derived growth factor (PDGF) signaling by oxidizing the PDGF receptor-β and enhancing downstream signaling [13,15]. Increased PDGF signaling has been linked to augmented cell survival and proliferation in many cancers [16]. Accordingly, PDGF-BB acts as an initiator for mitotic proliferation in MKs when it binds to PDGF receptor-β. In PMF, the oxidation of PDGF receptor-β by LOX causes increased binding to PDGF-BB and leads to further expansion of the MK lineage [11]. Given that immature MKs produce LOX at much higher levels than mature MKs, this process of PDGF receptor-β modification creates a feed-forward loop which further contributes to the over-proliferation of MKs in PMF [11].

### LOX Inhibitors and Their Potential Therapeutic Advantages

The role of LOX in BM fibrosis makes it a potential therapeutic target. Beta-aminopropionitrile (BAPN) is a small molecule, pan-LOX inhibitor that performs irreversible inhibition of LOX by forming a complex with its active site [17,18]. Due to this irreversible inhibition, BAPN is a potent LOX inhibitor. BAPN was administered to a Gata-1 low mouse model of PMF, a model characterized by increased MK number and a fibrotic BM due to a mutation in the GATA-1 transcription factor [11,19]. GATA-1 controls the terminal maturation of MKs, and in its absence or reduced expression, MKs undergo increased proliferation [19]. In this study, BAPN treatment slowed the development of the myelofibrotic phenotype [11]. Through the inhibition of LOX by BAPN, the activity of LOX in the ECM was decreased as well as its impact on PDGF receptor-β. By decreasing PDGF signaling, BAPN treatment significantly reduced the number of MKs present in the BM, but did not affect their overall ploidy [11]. Given that LOX is produced by immature MKs, this reduction in MK number along with the inhibition of LOX cross-linking activity in the ECM made BAPN effective in attenuating the myelofibrotic phenotype of the mice [11]. This was a significant early study in demonstrating the potential benefit of LOX inhibition in the progression of PMF, and provided a novel therapeutic strategy by specifically targeting ECM dysregulation. However, BAPN is a non-specific inhibitor of other amine oxidases and has the potential of creating off-target effects which generate reactive oxygen species that can cause organ damage [17,20]. These hazards limit the utility of BAPN as a therapy for PMF, but nonetheless opened the door for future studies using LOX-specific inhibitors. Two such inhibitors, PXS-LOX_1 and PXS-LOX_2, are novel haloallylamine-based inhibitors capable of targeting lysyl oxidases with a high degree of specificity [21–23]. Both PXS-LOX_1 and PXS-LOX_2 are promising not only because of their specificity for lysyl oxidases, but because of their similar potency to BAPN [24]. They have been shown to be mechanism-based inhibitors based on the classical tests for irreversibility and time-dependent enzyme inactivation. These characteristics made PXS-LOX_1 and PXS-LOX_2 promising candidates as potential therapeutics.

### PXS-LOX_1 Treatment Reduces BM Fibrosis and MK Number in GATA-1 Low Mice

To test the impact of PXS-LOX_1 on the pathogenesis of PMF, it was given to GATA-1 low mice. As noted above, the GATA-1 low model is advantageous for the study of PMF because the progression of the myelofibrotic phenotype mirrors that of humans, including an increased number of immature MKs, BM fibrosis, and splenomegaly [11,19,25]. PXS-LOX_1 was administered via injection at a dose of 15 mg/kg four times a week for nine weeks before the mice were sacrificed for analysis, where the spleen and femurs were harvested [24]. Injections began when the mice were 15 to 16 weeks of age, when fibrosis is expected to initiate [26,27]. While the tested mice maintained the same weight and blood count (with the exception of a small reduction in platelet number towards the end of the testing period) compared with the vehicle-treated control mice, the extent of BM fibrosis had been significantly reduced. The level of fibrosis was blindly quantitated using several bone marrow grids, with the subsequent comparison showing a significant decrease in fibrosis in the PXS-LOX_1-treated mice [28]. Of note, this experiment was conducted on both male and female mice, and while both genders showed the same level of BM fibrosis when treated with PXS-LOX_1, the female vehicle-treated control mice showed significantly greater BM fibrosis than the males. In addition to BM fibrosis, splenomegaly is another aspect of PMF that contributes to the pain and debilitating symptoms associated with the disease, and reduction of spleen size is often used as a clinical end point in PMF clinical trials [29,30]. After the normalization of spleen size to body weight, a trend appeared showing that PXS-LOX_1 caused a decrease in spleen size compared to the vehicle-treated controls [24]. Another hallmark of PMF, an overabundance of immature MKs, was also partially mitigated by PXS-LOX_1. The number of MKs followed the same trend as the degree of BM fibrosis described above: female vehicle-treated control mice had higher MK counts than their male counterparts, while both male and female PXS-LOX_1-treated mice showed a similar MK count to one another, and a significant reduction in MKs compared to the vehicle-treated controls [24]. This trend makes sense given that in PMF, the over-abundance of LOX enhances PDGF signaling and its mitogenic effect by oxidizing the PDGF receptor.

### PXS-LOX_2 Treatment Decreases BM Fibrosis in JAK2V617F Mice, but Decreased MK Number and Spleen Size Was Not Observed in Females

Similar to the PXS-LOX_1 result, administration of PXS-LOX_2 began when the mice were 15–17 weeks of age [26,27]. Unlike PXS-LOX_1, PXS-LOX_2 was tested on Vav1-hJAK2V617F (JAK2V617F) mice [24]. This humanized mouse line also displays the hallmarks of PMF including an enlarged spleen, an expansion of the MK lineage, and BM fibrosis [26]. Notably, the JAK2V617F mutation of this mouse line is the same mutation present in 60% of human PMF cases and displays the phenotype of early PMF including panmyelosis and leucoerythroblastic blood smear. PXS-LOX_2 was given orally at a dose of 5 mg/kg, once a day four times a week for a total of 8 weeks [24]. Treatment with PXS-LOX_2 was confirmed to result in a reduction in LOX activity and had no discernable impact on blood count, but caused a reduction in body weight (of about 10%) in treated mice when compared to controls [24]. Vehicle-treated control and PXS-LOX_2-treated WT mice showed no difference in reticulin fibrosis. As for the JAK2V617F mice, treatment with PXS-LOX_2 reduced reticulin fibrosis by 45.7% in male mice and by 90.0% in female mice compared to their respective vehicle-treated controls [24]. However, the JAK2V617F mice all displayed reticulin at least in two orders of magnitude greater than the WT mice. Interestingly, the trend of the female JAK2V617F mice being more impacted by the treatment was seen. Spleen size was reduced significantly for treated females compared to the vehicle-treated controls, but there was no noticeable reduction in males [24]. Additionally, while it was previously stated that PXS-LOX_2 treatment reduced reticulin fibrosis in both males and females, treated females also saw a 61.5% reduction in reticulin level compared to the vehicle-treated control mice while treated males again displayed no noticeable reduction in this property [24]. As for the MK number, treated JAK2V617F females showed a reduction in their MK number while treated males were unaffected. It is speculated that this sex dependent variability is due to the fact that the onset of fibrosis is faster and more severe in male JAK2V617F mice compared to females [24].

The results of PXS-LOX_2 being more effective on female JAK2V617F mice than males raised the question of whether PXS-LOX_1, which was equally effective in male and female GATA-1low mice, would maintain its effectiveness in male JAK2V617F mice. To answer this, the PXS-LOX_1 procedure was modified so that it would be given orally to remain consistent with the procedure of the PXS-LOX_2 test, and was administered at a dose of 30 mg/kg four times a week for eight weeks [24]. Both the treated mice and vehicle-treated controls displayed similar changes in weight, platelet levels, and hemoglobin levels. The treated mice displayed a 76.8% reduction in fibrosis compared to the vehicle-treated controls, showing that PXS-LOX_1 could effectively attenuate fibrosis in male JAK2V617F mice [24]. However, there was no difference in spleen size between the two groups, meaning that PXS-LOX_1 was not as effective in JAK2V617F mice as it had been in GATA-1 low mice. Regardless, the impact of these novel inhibitors on the progression of PMF shows that LOX is a key factor the dysregulation of the ECM, and a worthwhile therapeutic target.

## Discussion: Potential Therapeutic Advantage of LOX Specific Inhibitors PXS-LOX_1 and PXS-LOX_2

PMF has few treatment options, with most of them being palliative in nature. The only curative treatment is an allogenic hematopoietic stem cell transplant, for which few patients are eligible. The available therapies to help ease the progression of PMF include treatments with significant side effects such as with ruxolitinib. While ruxolitinib is effective in improving splenomegaly, and has been shown to modestly improve long-term survival of patients, it also causes anemia, cytopenias, and withdrawal syndrome (acute relapse of PMF symptoms, splenomegaly and septic shocklike symptoms) [29–31]. The harsh side effects of available drugs like ruxolitinib paired with the lack of any therapeutic that specifically targets ECM dysregulation of PMF highlights the potential benefits of PXS-LOX_1 and PXS-LOX_2. As stated previously, the studies using BAPN showed that the inhibition of LOX was a potential avenue of treatment for PMF [11]. While BAPN’s lack of specificity limited its utility as a therapeutic, its effectiveness in attenuating BM fibrosis prompted the development of LOX inhibitors with increased specificity [17,20]. One such inhibitor is PXS-5055, which reduced LOX and LOXL-2 activity when administered to myelofibrosis patients that had developed a resistance to ruxolitinib in a phase 1 trial [32]. This trial demonstrated the potential of LOX inhibitors to be impactful therapeutics, and, in addition to PXS-LOX_1 and PXS-LOX_2, more inhibitors such as PXS-S2B and PXS-5153A are currently under development [24,33]. What differentiates these pan-lysyl oxidase inhibitors from one another is their specificity. For instance, PXS-LOX and PXS-LOX_2 are most effective in inhibiting LOX, PXS-S2B primarily targets LOXL-2, and PXS-5153A inhibits both LOXL-2 and LOXL-3 [24,33]. This difference in specificity opens the possibility for future prescription of treatment that could target a specific LOX or LOX-like enzyme.

As mentioned earlier, the differences in effectiveness due to sex seen in the PXS-LOX_2 tests may be due to the less severe onset of fibrosis in female mice. This is a trend seen in human patients as well. Of patients with MPNs, males were more likely to present with PMF than females (females were more likely to present with essential thrombocytosis). Males with MPNs also display inferior survival compared to female patients [34]. A potential explanation for the increased survival seen in females is due to a difference in estrogen levels. Estrogen receptor-β (ERβ) is known to be expressed by MKs and plays a role in megakaryocytopoiesis [35,36]. In fact, ERβ is capable of binding directly to the promoter of GATA-1 and activate its transcription [37]. Given that GATA-1 regulates the development of MKs, the higher estrogen levels in females may help to maintain increased MK differentiation, therefore dampening the severity of the onset of PMF.

Increasing our understanding of how the dysregulation of the ECM affects the progression of PMF will provide novel avenues for treatment, and the current lack of treatment options for this aspect of PMF means that further study on these drugs is of great interest. LOX inhibitors would likely be most effective when administered to patients with pre-fibrotic PMF because of their ability to mitigate the progression of fibrosis [24]. The World Health Organization defines “pre-PMF” as megakaryocytic proliferation and atypia without reticulin fibrosis, as well as splenomegaly, leukocytosis, anemia and the presence of the known driver mutations [38]. The ability to employ LOX inhibitors to help delay the onset of fibrosis could be a major development given that there is currently no FDA approved treatment for pre-PMF.

## Abbreviations:

BAPN: Beta-aminopropionitrile
BM: Bone Marrow
ECM: Extra Cellular Matrix
ERβ: Estrogen Receptor β
JAK2V617F: Vav1-hJAK2V617F
LOX: Lysyl Oxidase
MK: Megakaryocyte
MPN: Myeloproliferative Neoplasm
PDGF: Platelet Derived Growth Factor
PMF: Primary Myelofibrosis
